# Quercetin induced apoptosis in association with death receptors and fludarabine in cells isolated from chronic lymphocytic leukaemia patients

**DOI:** 10.1038/sj.bjc.6605794

**Published:** 2010-07-20

**Authors:** M Russo, C Spagnuolo, S Volpe, A Mupo, I Tedesco, G-L Russo

**Affiliations:** 1 Institute of Food Sciences, National Research Council, Avellino 83100, Italy; 2 Onco-Haematology Division, SG Moscati Hospital, Avellino 83100, Italy

**Keywords:** chronic lymphocytic leukaemia, apoptosis, TRAIL, CD95, fludarabine, quercetin

## Abstract

**Background:**

Quercetin is a flavonoid naturally present in food and beverages belonging to the large class of phytochemicals with potential anti-cancer properties. Here, we investigated the ability of quercetin to sensitise primary cells from chronic lymphocytic leukaemia (CLL) to death receptor (DR) agonists, recombinant TNF-related-apoptosis-inducing ligand (rTRAIL) and anti-CD95, and to fludarabine, a widely used chemotherapeutic drug against CLL.

**Methods:**

Peripheral white blood cells were isolated from patients and incubated with medium containing 50 ng ml anti-CD95 agonist antibody; 10 ng ml recombinant TRAIL; 10–25 *μ*M quercetin and 3.5–14 *μ*M fludarabine. Neutral Red assay was used to measure cell viability, where as apoptosis was assessed by determining caspase-3 activity, exposure to Annexin V and PARP fragmentation.

**Results:**

Quercetin significantly enhanced anti-CD95- and rTRAIL-induced cell death as shown by decreased cell viability, increased caspase-3 and -9 activities, and positivity to Annexin V. In addition, association of quercetin with fludarabine increases the apoptotic response in CLL cells of about two-fold compared with quercetin monotreatment.

**Conclusion:**

This work shows that resistance to DR- and fludarabine-induced cell death in leukaemic cells isolated from CLL patients can be ameliorated or bypassed by the combined treatment with quercetin. Considering the low toxicity of the molecule, our study results are in favour of a potential use of quercetin in adjuvant chemotherapy in combination with other drugs.

Chronic lymphocytic leukaemia (CLL) is the most frequent form of leukaemia in adult population (22–30% of all leukaemia cases) with an incidence projected to be between 1 and 5.5 per 100 000 people in the Western world (Yee and O’Brien, 2006). Approximately, 50% of CLL patients remain asymptomatic and are diagnosed by unrelated lymphocytosis. Treatment depends on the clinical staging (Rai or Binet classification) and, originally, included conventional therapy based on alkylating/anthracycline agents. The introduction in therapy of fludarabine, a purine analogue, substantially improved overall and complete responses when used both as monotherapy (Johnson *et al*, 1996) and in association with alkylating agents (Eichhorst *et al*, 2006). Recently, treatment with humanised monoclonal antibodies, such as rituximab and alemtuzumab has been used as induction therapy and in patients with refractory CLL (Yee and O’Brien, 2006). In these patients, modest improvement has been achieved with rituximab or combined chemotherapy. Despite this, a significant percentage of untreated patients (up to 37%) do not respond to fludarabine treatment, or become refractory to this regime of treatment (up to 76%). Moreover, a variable number of these subjects occasionally incur in severe side effects, such as neutropenia, suppression of helper T cells, acute tumour lysis syndrome, autoimmune haemolysis, and neurotoxicity (Yee and O’Brien, 2006).

Cell death evasion and progressive accumulation of B cells are the major relevant events in CLL pathogenesis, so targeting apoptotic pathways triggered by CD95 (Fas/Apo-1) and TNF-related-apoptosis-inducing ligand (TRAIL)/Apo-2 death receptors (DRs) has been suggested as novel therapeutic approaches to treat this leukaemia (Pleyer *et al*, 2009). CD95 and TRAIL belong to the TNF receptor superfamily regulating the extrinsic apoptotic pathways after the interaction with their cognate ligand. Both CD95 and TRAIL are potential candidates to target apoptosis in malignant cells (Debatin and Krammer, 2004; Papenfuss *et al*, 2008; Russo *et al*, 2010). However, one drawback of this therapeutic strategy is the resistance to DR-induced cell death in B-CLL patients (MacFarlane *et al*, 2002, 2005).

In this context, a plethora of naturally occurring molecules with chemopreventive properties have been suggested as potential candidates in adjuvant chemotherapy when associated with other drugs (Aggarwal and Shishodia, 2006; Mansour *et al*, 2007; Russo, 2007; Fimognari *et al*, 2008). In this article, we show that quercetin (3,3′,4′,5,7-pentahydroxyflavone), a naturally occurring flavonoid widely present in fruits and beverages (Lamson and Brignall, 2000; Day *et al*, 2003) and possessing anti-cancer activity (Russo, 2007; Bischoff, 2008; Murakami *et al*, 2008), is able to sensitise leukaemic cells isolated from CLL patients when associated with recombinant TRAIL (rTRAIL) or anti-CD95 agonistic antibody. We also showed that quercetin potentiates the effect of fludarabine on resistant B-CLL cells. The rationale for investigating the sensitising effect of quercetin on CLL derives from previous works on leukaemic cell lines showing the apoptogenic activity of the molecule on cell lines resistant to DR-induced cell death (Russo *et al*, 1999, 2003, 2007). To the best of our knowledge, this is the first study to show the anti-cancer and apoptogenic effects of quercetin at relatively low concentrations (10-25 *μ*M) in primary tumour cells.

## Materials and methods

### Reagents

Roswell Park Medium Institute (RPMI) medium, L-glutamine 200 mM, penicillin 5000 IU ml^−1^/streptomycin 5000 *μ*g ml^−1^ and phosphate-buffered saline (PBS) tablets were purchased from Invitrogen (S Giuliano Milanese, Milan, Italy). Neutral red 0.33% solution, propidium iodide, Trypan blue solution (0.4%), quercetin, and dimethyl sulfoxide (DMSO) were from Sigma-Aldrich (Milan, Italy). Annexin V-FITC, rTRAIL (super killer TRAIL), caspase-3 and -9 substrates were from Enzo Life Sciences (AG Lausen, Switzerland). Anti-CD95 (clone CH-11) was from Immunotech (Marseille, France). Fludarabine phosphate was gifted by Onco-Haematology Division, SG Moscati Hospital (Avellino, Italy), participating in this study.

### Cell isolation and viability tests

Mononuclear cells (leukaemic cells >90%) were isolated from peripheral blood of 47 patients with B-CLL. All clinical samples were obtained after informed consent. Following density-gradient centrifugation (Ficoll-Paque Plus; GE Healthcare, Milan, Italy), cells were washed three times in PBS, counted with Trypan blue dye to assess their viability (cell viability >95%), and were immediately cultured in RPMI supplemented with 1% penicillin/streptomycin, 2 mM L-glutamine and 10% autologous serum (Bomstein *et al*, 2003), at 37°C in a humidified atmosphere containing 5% CO_2_. For neutral red assay (Fautz *et al*, 1991), we cultured the cells at density of 1 × 10^6^ per ml in 48 multi-well plates and incubated (24–48 h) in a medium containing 0.1% DMSO, 10–25 *μ*M quercetin solubilised in 0.1% DMSO, rTRAIL (10 ng ml^−1^), anti-CD95 (50 ng ml^−1^), or fludarabine dissolved in PBS (3.5–14 *μ*M final concentration). Cell viability assay was performed as described (Russo *et al*, 2003).

### Apoptosis assays

Phosphatidylserine exposure was measured using the binding of fluorescein-isothiocyanate-labelled (FITC) Annexin V to phosphatidylserine, as per the manufacturer’s protocol (Enzo Life Sciences). Briefly, cells (2 × 10^6^ per ml) were washed in PBS and suspended in binding buffer (10 mM HEPES (pH 7.4); 140 mM NaCl; 2.5 mM CaCl_2_). Annexin V FITC (5 *μ*l) and propidium iodide (10 *μ*l) were added to the cells for 10–15 min in the dark at room temperature and analysed with flow cytometer (FACSCalibur; Becton Dickinson, Mountain View, CA, USA) equipped with argon laser (488 nm) and filtered at 530 and 585 nm for FITC and phycoerythrin respectively. Low fluorescence debris and necrotic cells, permeable to propidium iodide, were gated out before to analysis and 10–20 000 events were collected. Data were analysed using CellQuest software (Becton Dickinson). This assay was also used to measure spontaneous apoptosis in freshly isolated B-CLL cells.

For caspase-3 and -9 enzymatic activities, we incubated cells (2 × 10^6^ per ml) for 12–16 h as described above for the cell viability test. At the end of incubation, cells were washed twice in PBS and suspended in lysis buffer (10 mM HEPES (pH 7.4), 2 mM ethylenediaminetetraacetic acid, 0.1% 3-((3-cholamidopropyl)dimethylammonio)-1-propanesulfonate, 5 mM dithiothreitol, 1 mM phenylmethylsulfonylfluoride, 10 *μ*g ml^−1^ pepstatin-A, 10 *μ*g ml^−1^ apronitin, 20 *μ*g ml^−1^ leupeptin). Following measurement of protein concentration (Bradford, 1976), cell extracts were added with reaction buffer and the respective conjugated amino-4-trifluoromethyl coumarin (AFC) substrates, for example, benzyloxycarbonyl-Asp(OMe)-Glu(OMe)-Val-Asp(OMe)-AFC(Z-DEVD-AFC)for caspase-3 and LEHD-AFC for caspase-9 (carbobenzoxy-Asp-Glu-Val-Asp and Leu-Glu-Hys-Asp-AFC) before incubation at 37°C for 30 min. Upon proteolytic cleavage of the substrates by the different caspases, the free fluorochrome AFC was detected by a spectrofluorometer multiplate reader (FL-500; Bio-Tek Instruments, Milan, Italy) with excitation and emission setting of 395±20 and 530±20 nm respectively. To quantify enzymatic activities, we determined an AFC standard curve. Caspase-specific activities were calculated as nmol of AFC produced per min per *μ*g proteins at 37°C at saturating substrate concentrations (50 *μ*M) (Russo *et al*, 2003). Fold increase in caspase-3 and -9 activities was determined by direct comparison with the level of DMSO-treated cells.

### Immunoblot

Cleavage of poly(ADP-ribose) polymerase (PARP) in B-CLL cells was revealed by immunoblotting using anti-PARP monoclonal antibody (Santa Cruz Biotechnology, Heidelberg, Germany) as described (Russo *et al*, 2007). B-CLL cells (2 × 10^6^ per ml) were suspended in lysis buffer containing 150 mM NaCl, 50 mM Tris-HCl (pH 7.4), 5 mM ethylenediaminetetraacetic acid, 1% NP-40, 0.5 mM dithiothreitol, 1 mM Na_3_VO_4_, 40 mM NaF, 1 mM Na_4_P_2_O_7_, 7.4 mg ml^−1^ 4-*p*-nitrophenyl phosphate, 10% glycerol, 100 *μ*g ml^−1^ phenylmethylsulfonyl fluoride and a cocktail of inhibitors ‘complete’ by Roche Applied Science (Monza, Milan, Italy).

Total protein lysates (20–25 *μ*g) were loaded on a 12% pre-cast gel (CRITERION XT; Bio-Rad Laboratories, Segrate, Milan, Italy) and blotted onto polyvinylidene difluoride (PVDF), hybond-P membrane (GE Healthcare). The membrane blot was rinsed with T-TBS (0.1% Tween 20, 25 mM Tris, 137 mM NaCl, 2.69 mM KCl (pH 8)) and blocked by 5% (w/v) non-fat dry milk in T-TBS for 1 h at room temperature. Subsequently, the membrane was incubated for 16 h at 4°C with anti-PARP antibody and finally incubated with anti-mouse horseradish-peroxidase-linked secondary antibody (GE Healthcare). The immunoblots were developed using Western Lightening Chemiluminescence Reagent Plus (PerkinElmer; Monza, Milan, Italy).

## Results

We first showed that quercetin enhances DR-induced cell death in leukaemic cells resistant to apoptotic stimuli (Russo *et al*, 1999, 2007); subsequently, others confirmed our original observation on cell lines of different origin (Psahoulia *et al*, 2007; Kim *et al*, 2008a, 2008b; Siegelin *et al*, 2009). However, to date, the pro-apoptotic effects of quercetin have not been investigated in cells isolated from primary tumours. The rationale of this work was to show if the sensitising effect of quercetin towards DR-induced cell death was confirmed in B cells isolated from CLL patients. To this aim, we isolated mononuclear cells (leukaemic cells >90%) from peripheral blood of 47 patients with CLL whose clinical features are reported in Table 1. The diagnosis of CLL was established by examination of the blood and bone marrow, including immunophenotype of the malignant lymphocytes according to IWCLL criteria (Hallek *et al*, 2008). These patients were either untreated or had not received treatment during the previous 6 months. The median age at diagnosis was 62 years (range 32–80) with a male to female ratio of 1 : 1. At diagnosis the distribution of Rai stages I, II, III, and IV was 0, 57, 2, and 41% respectively. Several molecules that modify B-cell receptor signalling, such as ZAP-70 or CD38, as well as immunoglobulin variable heavy chain gene mutation status show important prognostic power in B-CLL at early clinical stage (Pleyer *et al*, 2009). Table 1 reports positivity to CD38, which is associated to both survival and proliferation in B-CLL cells. CD38 expression and signalling capacity are also linked to progression and response to therapy (Damle *et al*, 1999). However, in this study, no statistical correlation has been measured between enhanced CD38 expression and cell death induced by quercetin treatment (Supplementary Figure S1).

Owing to the well-known heterogeneity of B-CLL cells, a preliminary screening was performed to determine sensitivity to quercetin, anti-CD95, and rTRAIL (data not reported). This analysis allowed us to address two essential points: (1) to confirm that all B-CLL samples were partially or totally resistant to anti-CD95 or rTRAIL treatments, accordingly to data reported in the literature previously (MacFarlane *et al*, 2002, 2005); (2) to establish the correct range of quercetin concentrations to avoid cytotoxic doses of quercetin that could mask the ability of the molecule to sensitise B-CLL cells to DR-dependent apoptosis. Therefore, in this arm of the study, we excluded samples extremely sensitive to quercetin (decrease in cell viability higher than 30% data not shown) and focused our analyses on cells resistant to DR-induced apoptosis (indicated by footenote d in Table 1).

**Figure 1:**
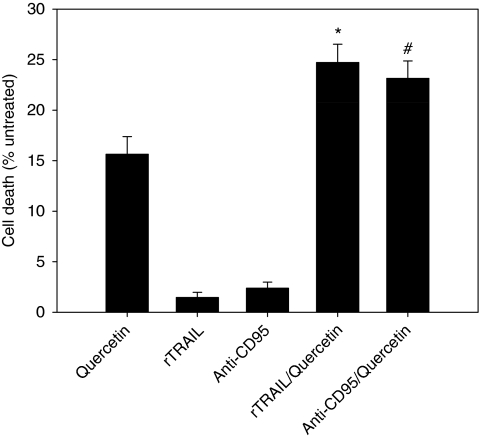
Cell viability assay in cells isolated from CLL patients and treated with quercetin and anti-CD95 or rTRAIL. Concentrations are as reported in Table 2. Values are presented as mean±s.e. (*n*=29) and significant differences were calculated using the two-tailed paired *t*-test. Asterisk (^*^) indicates significant difference from quercetin (*P*<0.001) and rTRAIL (*P*<0.0001), whereas (#) indicates significant difference from quercetin (*P*<0.001) and anti-CD95 (*P*<0.0001).

Cell viability assays (Figure 1) indicate that quercetin potentiates sensitivity to anti-CD95 and rTRAIL treatment with an increase in death cells of about 1.5- and 1.6-fold respectively, when compared with quercetin monotreatment. This effect is statistically significant, as indicated in Figure 1, and suggests the presence of two parallel events acting on B-CLL cells, both triggered by quercetin: (1) sensitisation to DR-induced apoptosis, and (2) apoptotic/cytotoxic effect of quercetin *per se*. It is noteworthy that cell viability assay (neutral red) applied to data presented in Figure 1 measured dead cells, without discriminating between apoptosis and necrosis. Therefore, to confirm that the combined treatment of quercetin in association with anti-CD95/rTRAIL triggers apoptotic pathways in B-CLL cells reported in Figure 1, we assayed canonical markers of DR-induced apoptosis in selected samples (Table 2). Reduced cell viability parallels with increased caspase-3 activity and increased percentage of cells positive to Annexin V, a canonical marker of apoptotic events. Cases reported in Table 2 have been selected based on several criteria: (1) to show that the effects of quercetin in enhancing DR-induced cell death in terms of decrease in cell viability, caspase-3 activation, and Annexin V positivity are more than additive (arrows), supporting the apoptogenic sensitising capacity of quercetin (CLL-55, 56, 44, and 11); (2) to show that a limited percentage of samples (about 10%) isolated from B-CLL patients neither respond to quercetin, nor to the combined treatment (CLL-42), even if a transient increase in caspase-3 was detected. Probably, the measured activation of caspase-3 was not sufficient to exceed the minimal threshold necessary to trigger apoptosis; (iii) to highlight that in about 60–70% of CLL samples studied, quercetin *per se* induces apoptosis, as indicated by decreased cell viability and increased caspase-3 activity and Annexin V positivity (CLL-55, 44, and 11). In the remaining cases, an example is CLL-56, the molecule is simply cytotoxic or triggers caspase-independent events, as evidenced by a limited caspase-3 activity and Annexin V positivity.

**Figure 2:**
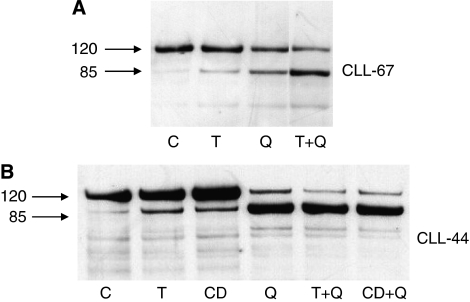
Cleavage of PARP in cells isolated from CLL patients. Immunoblottings show the proteolytic cleavage of PARP by caspase-3 in two selected samples isolated from CLL patients (CLL-67 and CLL-44 in panels **A** and **B** respectively). Cells from CLL-44 were treated as indicated in Table 1, whereas sample CLL-67 was treated with 20 *μ*M quercetin and 10 ng ml^−1^ rTRAIL. C, Q, T, CD indicate DMSO and quercetin, rTRAIL, anti-CD95 treated cells. Numbers on the left indicate the molecular weight of uncleaved (120 kDa) and cleaved (85 kDa) PARP. Images are representative of one experiment out of two performed for each sample.

The apoptogenic effect of quercetin associated with anti-CD95/rTRAIL has been also confirmed by the degradation of PARP. Demonstrative examples are shown in Figure 2. In the case of sample CLL-67 (panel A), the combined treatment (quercetin along with rTRAIL) increases PARP degradation compared with single treatments, whereas in the second case (panel B, corresponding to CLL-44), the apoptotic effect of quercetin monotreatment is prevalent, as shown in Table 2 in terms of cell viability, caspase-3 activation and annexin V exposure.

**Figure 3:**
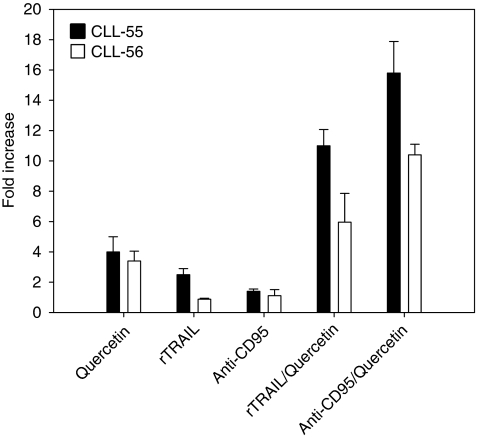
Caspase-9 activation in cells isolated from CLL patients. The proteolytic activity of caspase-9 (nmol AFC per min per *μ*g protein) was measured in two selected samples isolated from CLL patients (CLL-55 and CLL-56). Cells were treated as indicated in Table 2. Enzymatic activity was reported as -fold increase compared with DMSO-treated cells after 12 h stimulation. Bar graphs represent the mean of two experiments (±s.e.).

In selected samples, which showed an enhancing effect of quercetin associated with DR agonists, we also measured the activation of caspase-9. These experiments were carried out to show the ability of quercetin to trigger the intrinsic apoptotic pathway, which is characterized by release of mitochondrial cytochrome *c* into the cytosol and procaspase-9 processing. In the cytosol, cytochrome *c* binds to apoptosis protease activation factor 1 and the resultant complex recruits caspase-9 leading to its activation and cleavage of downstream caspases (Kuida, 2000). As shown in Figure 3, quercetin monotreatment increases caspase-9 activity by 3.5- and 4-fold in CLL-56 and CLL-55 respectively. This effect was further enhanced in the combined treatments with anti-CD95 and rTRAIL (Figure 3), confirming data reported in Table 2. It is interesting to note that increased caspase-9 by quercetin monotreatment in CLL-56 did not match with a strong activation of caspase-3 and apoptotic induction (Table 2), which, in turn, was obtained only in the combined treatments.

**Figure 4:**
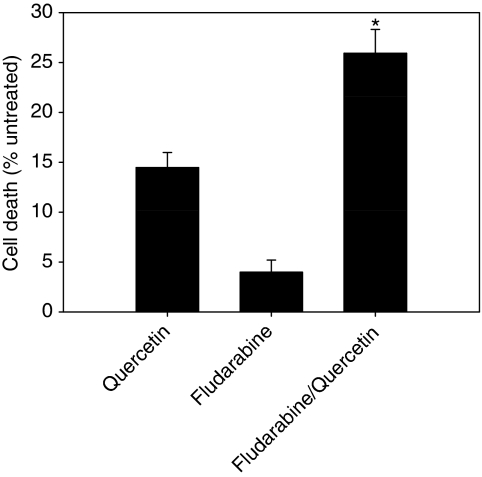
Cell viability assay in cells isolated from CLL patients and treated with quercetin and fludarabine. Range of concentrations used is as reported in Table 3. Values are presented as mean±s.e. (*n*=29) and significant differences were calculated using the two-tailed paired *t*-test. Asterisk (^*^) indicates significant difference from quercetin (*P*<0.0005) and fludarabine (*P*<0.0001).

In the majority of B-CLL cells investigated, quercetin monotreatment induced cell death at concentrations significantly lower than those showing cytotoxicity on cell lines of leukaemic origin (Russo *et al*, 2007; data not shown). On the basis of this observation, we reasoned that quercetin could also strengthen the efficacy of drugs widely used in the therapy of CLL, such as fludarabine (Yee and O’Brien, 2006). To test this possibility, we treated the samples isolated from B-CLL patients (indicated by footenote c in Table 1), showing a significant resistance to 3.5–14 *μ*M fludarabine, with quercetin in combination with fludarabine. It is noteworthy that 3.5 *μ*M represents the therapeutic plasma concentration of fludarabine (Binet, 1993). As reported in Figure 4, the association between quercetin and fludarabine increases cell death by approximately two-fold when compared with quercetin and by six-fold with respect to fludarabine. Differences were significant, as indicated in Figure 4. Selected samples listed in Table 3 show that the pro-apoptotic effects of the combined treatment are more than additive (arrows) with respect to the three markers used to assess cell death, suggesting the enhancing apoptogenic effect of quercetin also in association with fludarabine. The pleiotropic effect of quercetin on multiple cellular targets is exemplified in sample CLL-42, which is resistant to CD95-, TRAIL- (Table 2), and fludarabine-induced (Table 3) apoptosis. Quercetin is not able to bypass resistance to DR agonists, but ameliorates the sensitivity to fludarabine.

## Discussion

The development of high selective drugs triggering specific molecular target is the desirable and ambitious objective in leukaemia treatment. A suitable example is represented by the development of the tyrosine kinase inhibitor imatinib that has revolutionised the treatment of chronic myeloid leukaemia (Stegmeier *et al*, 2010). However, in the case of CLL, its heterogeneous nature and the resistance to conventional treatments developing in the majority of patients hinder the progresses in therapy. Our study opens new applicative perspectives supported by the observation that TRAIL receptor is preferentially expressed on malignant cells and appears a better candidate in cancer therapy and in B-CLL leukaemias, with respect to the high liver toxicity of anti-CD95 treatment (Igney and Krammer, 2002; MacFarlane *et al*, 2005).

Overall, this work shows that resistance to DR- and fludarabine-mediated cell death in leukaemic cells isolated from B-CLL patients can be ameliorated or bypassed by the combined treatment with quercetin. Similar data have been reported by our group in leukaemia cell lines resistant to DR-induced apoptosis (Russo *et al*, 1999, 2007). However, as mentioned above, this work represents, to the best of our knowledge, the first evidence that quercetin may potentiate apoptosis in cells isolated from primary tumours.

From data presented in Figures 1,2,3 and Table 2, we can hypothesise that in leukaemic cells isolated from B-CLL patients, the apoptotic response mediated by DRs could be inhibited, or strongly reduced, by unknown interfering mechanisms functioning on the extrinsic pathways. Quercetin is able to loosen this block potentiating DR-induced apoptosis in CLL cells. This implies the presence of a functional CD95 or TRAIL receptors. Only if this condition is verified quercetin can free the apoptotic pathways downstream CD95/TRAIL that are blocked or inhibited by anti-apoptotic factors. We also suggest that the moderate but significant enhancing effect of quercetin in inducing apoptosis when associated with anti-CD95, rTRAIL, and fludarabine (Figures 1 and 4) can be explained evoking the variability of B-CLL cells and the heterogeneity of the disease.

New combined therapeutic treatments are potential targets against resistant forms of B-CLL (Kaufmann and Steensma, 2005; Pleyer *et al*, 2009). The possibility that quercetin may ameliorate efficacy of chemotherapy is strengthen by the safety of the molecule administered through intravenous infusion. In fact, an extensive phase I clinical trial established that at dose of 10.8 mg kg^−1^ body weight, no adverse effects were observed (Ferry *et al*, 1996). The well-documented safety of quercetin (Okamoto, 2005; Harwood *et al*, 2007), together with the observation that quercetin does not induce apoptosis in human peripheral mononuclear cells (Lugli *et al*, 2009; Russo GL, Russo M, Spagnuolo C, data not shown), represents an important issue in favour of its potential use in combination with other therapeutic treatments, already approved and used in clinics.

The mechanism of action triggered by quercetin in CLL is still a matter of study. As partially shown here and in agreement with unpublished data from our laboratory on cell lines (Russo *et al*, in preparation), quercetin may exert its effect on both extrinsic and intrinsic apoptotic pathways. We confirmed this possibility in B-CLLs with the observation that quercetin triggers both DR- (extrinsic pathway) and fludarabine (intrinsic pathway)-induced cell death. The well-known antioxidant properties of the molecule are independent from its apoptogenic activity when associated with DRs or fludarabine. In fact, fluctuation of intracellular reactive oxygen species in B-CLL cells does not correlate with quercetin treatments and/or its cytotoxicity (Supplementary Figure S2). A similar behaviour was reported in leukaemic cell lines (Russo *et al*, 2003). We also explored other mechanism(s) of action possibly triggered by quercetin. One rationale hypothesis to explain the sensitising apoptotic effect of the molecule in rTRAIL-induced cell death could be its ability to stimulate the expression of TRAIL receptor(s). In this context, it is worthwhile to remember that pivotal works from Cohen's group showed that, in B-CLL, TRAIL-R1 is predominantly expressed over TRAIL-R2, whereas no putative decoy receptors, TRAIL-R3 and TRAIL-R4, are present on the surface of B-CLL cells (MacFarlane *et al*, 2002, 2005). Unfortunately, we showed that quercetin does not modify expression of TRAIL-R1 (Supplementary Figure S3). Alternatively, considering the importance of Bcl-2 family member in controlling apoptosis in B-CLL (Packham and Stevenson, 2005), we tested the ability of quercetin to interfere with Bcl-2 proteins regulation. Pro-apoptotic Bax and anti-apoptotic Bcl-xL cannot be considered important, functional targets of quercetin in B-CLL as their expression is extremely fluctuating in untreated B-CLL cells, probably due to the heterogenicity of the disease (Supplementary Figure S4A and B). In the case of pro-survival member Bcl-2, its expression appears more constant in B-CLL isolated from patients, but it is not influenced by quercetin treatment (Supplementary Figure S4C). Currently, we are screening other possible direct or indirect targets of quercetin that may be able to interfere with apoptotic pathways in cellular models resembling B-CLL.

A novelty of this study is the demonstration that quercetin was able to induce apoptosis in CLL samples in the absence of other treatments (Figures 1 and 4; Table 2 and 3). B cells isolated from patients were more sensitive to quercetin than cell lines of leukaemic origin, which showed neither cytotoxicity, nor apoptosis when treated with 50 *μ*M quercetin (Russo *et al*, 2007). In our view, this represents an important issue that stimulates further studies in the direction of therapeutic use of the molecule. Quercetin in CLLs may function as a double-blade knife combining its intrinsic apoptogenic activity with the ability to potentiate the effect of other chemotherapeutic treatments.

## Figures and Tables

**Table 1:** Clinical features of CLL patients

**Patient**	**Sex**	**Age**	**Stage (Rai)**	**CD38^+^^a^**	**WBC^b^**
CLL-69^c^	F	75	IV		68 400
CLL-67^d^	M	70	II		66 200
CLL-66^d^	M	80	IV	3	63 400
CLL-62	F	43	II	2	68 000
CLL-61^c^^,^^d^	F	62	II		70 000
CLL-60^c^^,^^d^	F	72	II	97	77 000
CLL-58^c^	F	75	II	3	65 000
CLL-57^c^	M	65	II	2	62 800
CLL-56^c^^,^^d^	M	68	II	20	72 000
CLL-55^c^^,^^d^	M	64	II	1	59 000
CLL-53	M	52	II		12 000
CLL-52^c^	F	49	II		80 100
CLL-51	M	72	IV	7	60 500
CLL-50	F	32	II	8	51 500
CLL-49^c^	M	57	IV	42	50 000
CLL-47	M	37	IV	3	66 900
CLL-46^c^^,^^d^	F	51	II	2	77 000
CLL-45^c^^,^^d^	M	64	II	1	59 900
CLL-44^c^^,^^d^	F	62	IV	60	67 200
CLL-43^c^^,^^d^	M	75	II	0	57 000
CLL-42^c^	M	78	IV		82 200
CLL-41^c^^,^^d^	F	65	IV	2	56 600
CLL-40^c^^,^^d^	F	66	IV	98	77 500
CLL-39^c^^,^^d^	M	62	IV	33	98 000
CLL-38^c^	F	72	II	6	66 500
CLL-35^d^	F	60	II		91 100
CLL-34^c^^,^^d^	F	60	II	74	50 000
CLL-33^d^	F	30	0		16 000
CLL-32^c^^,^^d^	M	66	II	0	63 000
CLL-30^d^	M	54	IV	6	46 000
CLL-29^c^^,^^d^	M	63	IV		56 300
CLL-28^c^^,^^d^	M	48	II	0	89 200
CLL-27	M	75	III	1	140 000
CLL-26^c^^,^^d^	M	65	II	10	62 000
CLL-25^c^^,^^d^	F	71	IV	15	65 900
CLL-24^c^^,^^d^	F	60	II	56	82 500
CLL-23^d^	M	56	II	78	57 900
CLL-22^c^^,^^d^	F	32	IV		135 000
CLL-20	M	62	II	6	85 900
CLL-19	F	74	IV	97	105 000
CLL-18^d^	M	62	IV	89	55 800
CLL-17	F	76	II	2	65 400
CLL-16	M	65	IV	0	49 200
CLL-15^c^^,^^d^	F	77	II	92	84 100
CLL-14^c^^,^^d^	F	63	II	1	65 400
CLL-12	M	59	II	0	80 000
CLL-11^c^^,^^d^	F	74	IV		40 000

Abbreviations: CLL=chronic lymphocytic leukaemia; M=male; F=female; WBC=white blood cell.

a Positivity to CD38. Accordingly to Schroers *et al* (2005) a 30% cut-off point was used; that is, the samples were considered CD38 positive if the antigen was present in 30% or more tumor cells and negative if expression was present in less than 30% cells.

b *n of* cells per *μ*l whole blood.

c Samples analysed in Figure 4.

d Samples analysed in Figure 1.

**Table 2:** Effects of quercetin on anti-CD95- and rTRAIL-induced apoptosis in cells isolated from CLL patients

**Patient**	**Treatment**	**Cell viability (%)**		**Caspase-3 (fold)**		**Annexin V (% positivity)**	
	Quercetin 10 *μ*M	86.7±0.9		1.4±0.3		3	
	rTRAIL 10 ng ml^−1^	101.5±0.9		1.4±0.4		3	
CLL 56	Anti-CD95 50 ng ml^−1^	97.0±1.5		1.6±0.4		0	
	rTrial+Quercetin	66.2±2.7	↓	5.6±0.4	↑	15	↑
	Anti-CD95+quercetin	77.7±0.69	↓	7.0±0.3	↑	10	↑
							
	Quercetin 10 *μ*M	72.1±2.3		3.7±0.25		15	
	rTRAIL 10 ng ml^−1^	97.2±3.7		0.79±0.01		5	
CLL 55	Anti-CD95 50 ng ml^−1^	106.9±5.5		1.39±0.15		1	
	rTrial+Quercetin	61.5±2.4	↓	4.6±0.43	↑	21	↑
	Anti-CD95+Quercetin	66.2±3.5	↓	5.5±0.15	↑	22	↑
							
	Quercetin 25 *μ*M	67±2.1		1.9±0.2		22	
	rTRAIL 10 ng ml^−1^	92.0±2.5		1.4±0.08		8	
CLL 44	Anti-CD95 50 ng ml^−1^	95.6±3.5		1.17±0.07		5	
	rTrial+Quercetin	55.5±3.3	↓	4.07±0.07	↑	33	↑
	Anti-CD95+Quercetin	55.6±1.8	↓	3.6±0.2	↑	35	↑
							
	Quercetin 25 *μ*M	92±3.2		2.1±0.1		6	
	rTRAIL 10 ng ml^−1^	114±8.5		2.0±0.06		0	
CLL 42	Anti-CD95 50 ng ml^−1^	101.4±2.0		1.3±0.06		3	
	TRAIL+Quercetin	103.2±3.7		1.9±0.2		6	
	Anti-CD95+Quercetin	107.3±3.5		2.0±0.06		5	
							
	Quercetin 25 *μ*M	77.5±3.6		2.1±0.05		20	
	rTRAIL 10 ng ml^−1^	90.5±8.5		1.2±0.16		7	
CLL 11	Anti-CD95 50 ng ml^−1^	95.5±2.0		0.77±0.11		12	
	rTRAIL+Quercetin	60±2.4	↓	4.1±0.44	↑	33	↑
	Anti-CD95+Quercetin	75.5±3.6	↓	2.6±0.2	↑	30	↑

Abbreviations: CLL=chronic lymphocytic leukaemia; rTRAIL=recombinant TNF-related-apoptosis-inducing ligand.

Arrows indicate that the decrease (↓) in cell viability, or the increase (↑) in caspase-3 activity and Annexin V positivity were more than additive with respect to single treatments with quercetin, anti-CD95, or rTRAIL. Percentage of Annexin V positivity was calculated subtracting basal values ranging between 10 and 30%.

**Table 3:** Cytotoxic effects of quercetin and fludarabine in cells isolated from CLL patients

**Patient**	**Treatment**	**Cell Viability (%)**		**Caspase-3 (fold)**		**Annexin V (% positivity)**	
	Quercetin 25 *μ*M	77.5±3.6		2.1±0.05		20	
CLL 11	Fludarabine 14 *μ*M	99±14.0		1.8±0.05		8	
	Quercetin+Fludarabine	23.5±1.2	↓	3.4±0.05		30	↑
							
	Quercetin 25 *μ*M	94.2±7.7		1.8±0.3		11	
CLL 41	Fludarabine 14 *μ*M	76.3±3.7		1.1±0.1		15	
	Quercetin+Fludarabine	66.8±0.9	↓	5.5±0.3	↑	35	↑
							
	Quercetin 25 *μ*M	92±3.2		2.1±0.1		6	
CLL 42	Fludarabine 14 *μ*M	96.4±4.0		2.5±0.13		6	
	Quercetin+Fludarabine	86±2.6	↓	5.1±0.2	↑	16	↑

Abbreviation: CLL=chronic lymphocytic leukaemia.

Arrows have the same meaning as in Table 2.
