# Supplementary material for: Quercetin induced apoptosis in association with death receptors and fludarabine in cells isolated from chronic lymphocytic leukaemia patients
Source: Br J Cancer. 2010 Jul 20;103(5):642–8. doi: 10.1038/sj.bjc.6605794 (PMC2938248; doi:10.1038/sj.bjc.6605794)
Supplement: Supplementary Materials [file 6605794x1.doc]

**Manuscript: TH/2010/4044**

***Supplementary material***

**Quercetin Induced Apoptosis in Association with Death Receptors and Fludarabine in Cells isolated from Chronic Lymphocytic Leukemia Patients**

M. Russo, C. Spagnuolo, S. Volpe, A. Mupo, I. Tedesco and G. L. Russo

**Figure S1. Correlation between CD38 expression and cell death induce by quercetin.**

Positivity to CD38 was determined as reported in legend of Table 1. Accordingly to previous work, (Schroers *et al*, 2005), a 30% cut-off point was used; that is, the samples were considered CD38 positive if the antigen was present in 30% or more tumor cells and negative if expression was present in less than 30% cells. Data points in the graph are referred to samples listed in Table 1 whose positivity to CD38 was above 30%. Cell death was determined by neutral red assay as reported in Materials and Methods section after treatment of B-CLL cells with quercetin (5-50 μM range). The two variables measured, e.g. cell death induced by quercetin and positivity to CD38, were uncorrelated (coefficient of determination R^2^ = 0.1386).

**Figure S2. ROS measurement in B-CLL cells treated with quercetin.**

ROS (Reactive Oxygen Species) were determined following standard protocols (Russo *et al*, 1999). Briefly, B-CLL cells (1.5x10^6^) isolated from patients indicated in Table 1 were incubated for 1 h in the presence of 25-50 μM of quercetin in RPMI complete medium as reported in Materials and Methods section. After washing, intracellular ROS concentration was determined incubating cells for 30 min in the presence of 20 μM DCFDA (2',7' dichlorofluorescein-diacetate) (Invitrogen). After washing, samples were spectofluorimetrically read with an excitation and emission setting of 495 and 530 nm, respectively. Cell death was determined by neutral red assay as reported in Materials and Methods section after treatment of B-CLL cells with quercetin (5-50 μM range) for 24 h and expressed as percentage of DMSO (0.1% v/v) treated cells. Bar graphs for cell death represent the mean of two independent experiments +s.e.

No correlation was evidenced between cell death induce by quercetin and its capacity to lower ROS. The two variables measured were uncorrelated (coefficient of determination R^2^ = 0.0114).

**
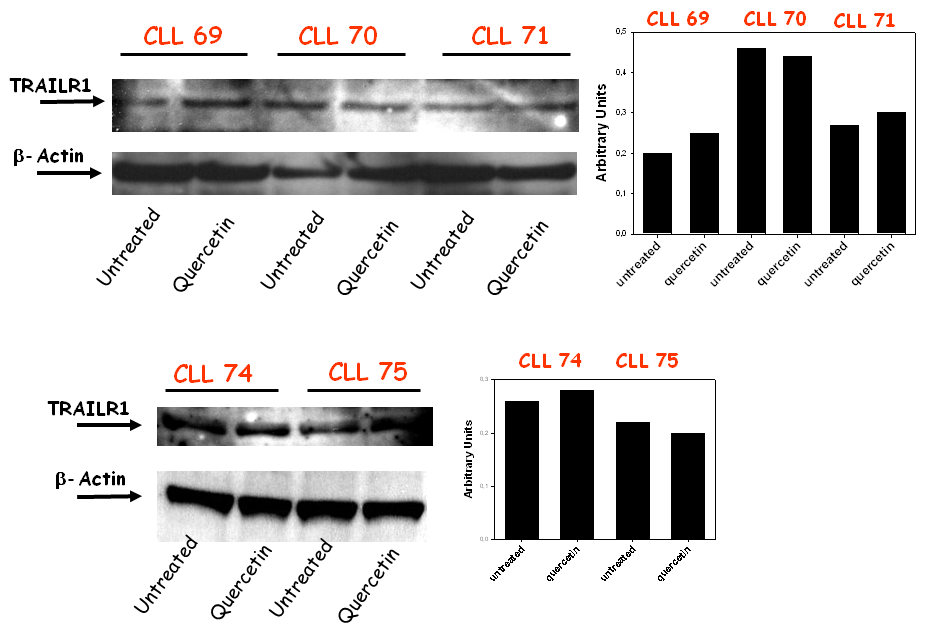
**

**Figure S3. Expression of TRAIL-R1/DR4 in B-CLL cells**

Immunoblotting shows the expression of TRAIL-R1/DR4 receptor in B-CLL cells isolated from five patients in the presence or absence of quercetin. Cells were treated with 0.1% DMSO (untreated control cells) or quercetin (20 μM) for 24 h as reported in Materials and Methods section. Subsequently, cells were lysed and cell extracts (30 μg) analysed by immunoblotting as described in Materials and Methods section. PVDF membranes were incubated 16 h at 4°C with anti-TRAIL-R1 polyclonal antibody (Santa Crutz Biotechnology, Heidelberg, Germany). After stripping, the same membrane was re-probed with an anti β-actin polyclonal antibody (Cell Signalling; Milan, Italy). Band intensity was quantitated measuring optical density on Gel Doc 2000 Apparatus (Biorad) and Multi-Analyst Software (Biorad). Images are representative of one experiment out of two performed for each sample.

Data in Figure S3 clearly shows that level of expression of TRAIL-R1 fluctuates among samples and quercetin treatment does not induce any significant increase in TRAIL-R1 expression. Therefore, we excluded that the sensitizing effect of quercetin in rTRAIL induced apoptosis could be associated to an increased number of receptors expressed on the cell membrane.

**
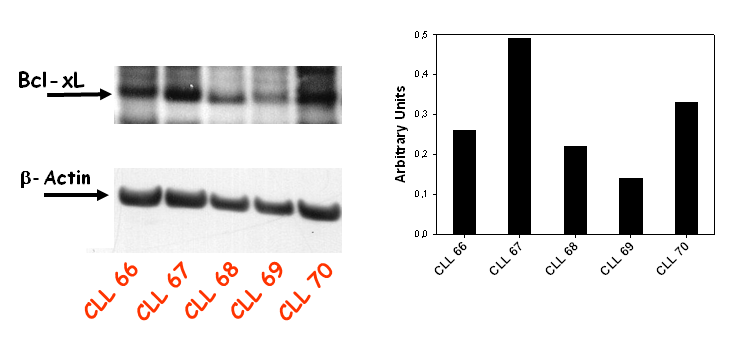
**

A

**
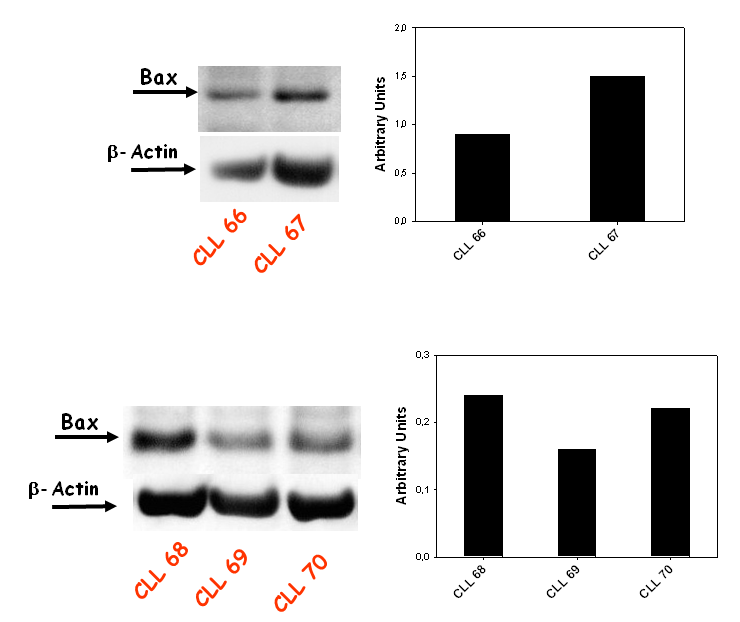
**

B

**
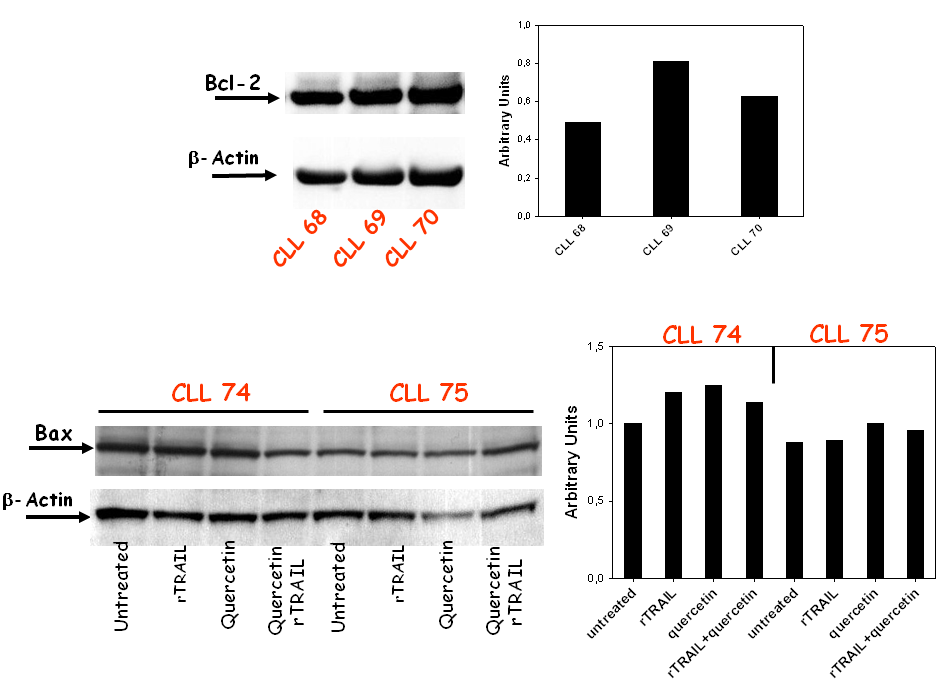
**

C

**Figure S4. Effect of quercetin on the expression of Bcl-2 family members (Bax, Bcl-xL, Bcl-2) in B-CLL cells**

Immunoblottings show the expression of Bcl-xL (panel A), Bax (panel B) and Bcl-2 (panel C) in B-CLL cells isolated from patients. For Bcl-xL and Bax, after immunoblotting, membranes were incubated 16 h at 4°C with anti Bcl-xL and anti-Bax polyclonal antibodies (Calbiochem **Merck Chemicals Ltd; Nottingham, UK**). For Bcl-2, three samples were analyzed only to detect the basal expression of Bcl-2, while in two cases, CLL-74 and CLL-75, cells were treated with 0.1% DMSO (untreated control), rTRAIL (10 ng/ml), quercetin (20 μM) and the association of rTRAIL plus quercetin for 24 h. After cell lysis and immunoblotting, membranes were incubated 16 h at 4°C with anti-Bcl-2 polyclonal antibody (Calbiochem). In all cases, membranes were re-probed with an anti β-actin polyclonal antibody (Cell Signalling). Band intensity was quantitated measuring optical density on Gel Doc 2000 Apparatus (Biorad) and Multi-Analyst Software (Biorad). Images are representative of one experiment out of two performed for each sample.

Data reported in Figure S4 clearly shows that the expression of Bcl-xL (A) and Bax (B) fluctuates excessively in untreated samples, probably due to the mentioned heterogenicity of the disease, making unlikely that the quercetin sensitizing effect of quercetin associated to CD95/TRAIL or quercetin may involve Bax or Bcl-xL.

Since expression of Bcl-2 appeared more constant among samples (C), in two cases, we treated B-CLL cells with quercetin, rTRAIL and their association to verify if quercetin was able to down-regulate Bcl-2 expression giving a rationale explanation to its enhancing apoptotic effect. As clearly reported for samples CLL-74 and CLL-75, Bcl-2 expression was not significantly inhibited by rTRAIL/quercetin treatment.

**References in supplementary material**

Russo M, Palumbo R, Tedesco I, Mazzarella G, Russo P, Iacomino G, Russo GL (1999) Quercetin and anti-CD95(Fas/Apo1) enhance apoptosis in HPB-ALL cell line. *FEBS Lett* **462**(3)**:** 322-8

Schroers R, Griesinger F, Trumper L, Haase D, Kulle B, Klein-Hitpass L, Sellmann L, Duhrsen U, Durig J (2005) Combined analysis of ZAP-70 and CD38 expression as a predictor of disease progression in B-cell chronic lymphocytic leukemia. *Leukemia* **19**(5)**:** 750-8
